# Hysterectomy Performed 52 Days After a Classic Cesarean Section Secondary to Uterine Myomas

**DOI:** 10.7759/cureus.105693

**Published:** 2026-03-23

**Authors:** Tanvi Karmarker, Diana Decotis-Smith, Mihir Waykar, Alysse Alejandro Hernandez, Steve Carlan

**Affiliations:** 1 Department of Obstetrics and Gynecology, Orlando Regional Medical Center, Orlando, USA

**Keywords:** classic cesarean section, incompetent cervix, preterm delivery, sepsis, uterine myomas

## Abstract

Uterine myomas complicating pregnancy can be a risk factor for cervical insufficiency, preterm birth, and cesarean delivery. Cervical insufficiency is a risk factor for preterm premature rupture of membranes (PPROM) and preterm births. A classic cesarean section (CS) is an incision made through the upper, thicker muscular segment of the uterus using a vertical uterine muscular incision and is at risk for postoperative infectious morbidity.

This report presents the case of a 32-year-old gravida 2 para 0 female who presented at 23 weeks and 5 days of gestation, 2 cm dilated, with PPROM. Prior to arrival, all of her prenatal care had been outside the United States and included an Arabin pessary. After a 15-day latency period from PPROM to the onset of labor, an emergency classic CS was performed, obtaining an 800-gram male infant with Apgar scores of 1 at 1 minute and 5 at 5 minutes. The infant expired on day of life 1. Over the next 52 days, she was admitted three times for clinical and radiologic findings consistent with recurrent infection and myoma degeneration. Her laboratory values remained stable as parenteral antibiotics were adjusted regularly. The patient requested maintaining her fertility throughout her care until her final admission. A total abdominal hysterectomy and bilateral salpingectomy were ultimately performed, and an intrauterine abscess, including the surgical site and degenerating myomas, was found. Serial imaging with both CT and ultrasound confirmed abnormal fluid density collections, but interventional radiology was unsuccessful in therapeutic drainage.

A preterm classic CS with myomas is associated with less favorable neonatal outcomes, higher complication rates, increased perioperative risk, and delayed recovery. Postoperative imaging could not differentiate between infectious causes and myoma degeneration in this case.

## Introduction

Preterm labor and premature preterm rupture of membranes (PPROM) are significant contributors to neonatal morbidity and mortality, particularly when occurring at the threshold of viability [1]. Management becomes even more complex in patients with cervical insufficiency, uterine anomalies, or fibroids [2]. The Arabin pessary is promoted as a non-surgical intervention for cervical insufficiency, though its use in high-risk patients remains controversial [3]. The presence of uterine fibroids, especially large, bulky, or pedunculated types, can significantly complicate pregnancy, delivery, and the puerperium, even without cervical insufficiency [4].

This case involves a patient with a complex perinatal course, including cervical insufficiency identified by physical examination and managed with an Arabin pessary. She had a subsequent PPROM and a technically challenging classic cesarean delivery attributable to uterine fibroids. The postpartum period was complicated by sepsis and recurrent infections and ultimately required a hysterectomy. Notably, this case features serial imaging of the abdomen and pelvis in the context of a postoperative uterus with myomatous degeneration and abscess formation.

## Case presentation

**Figure 1:**
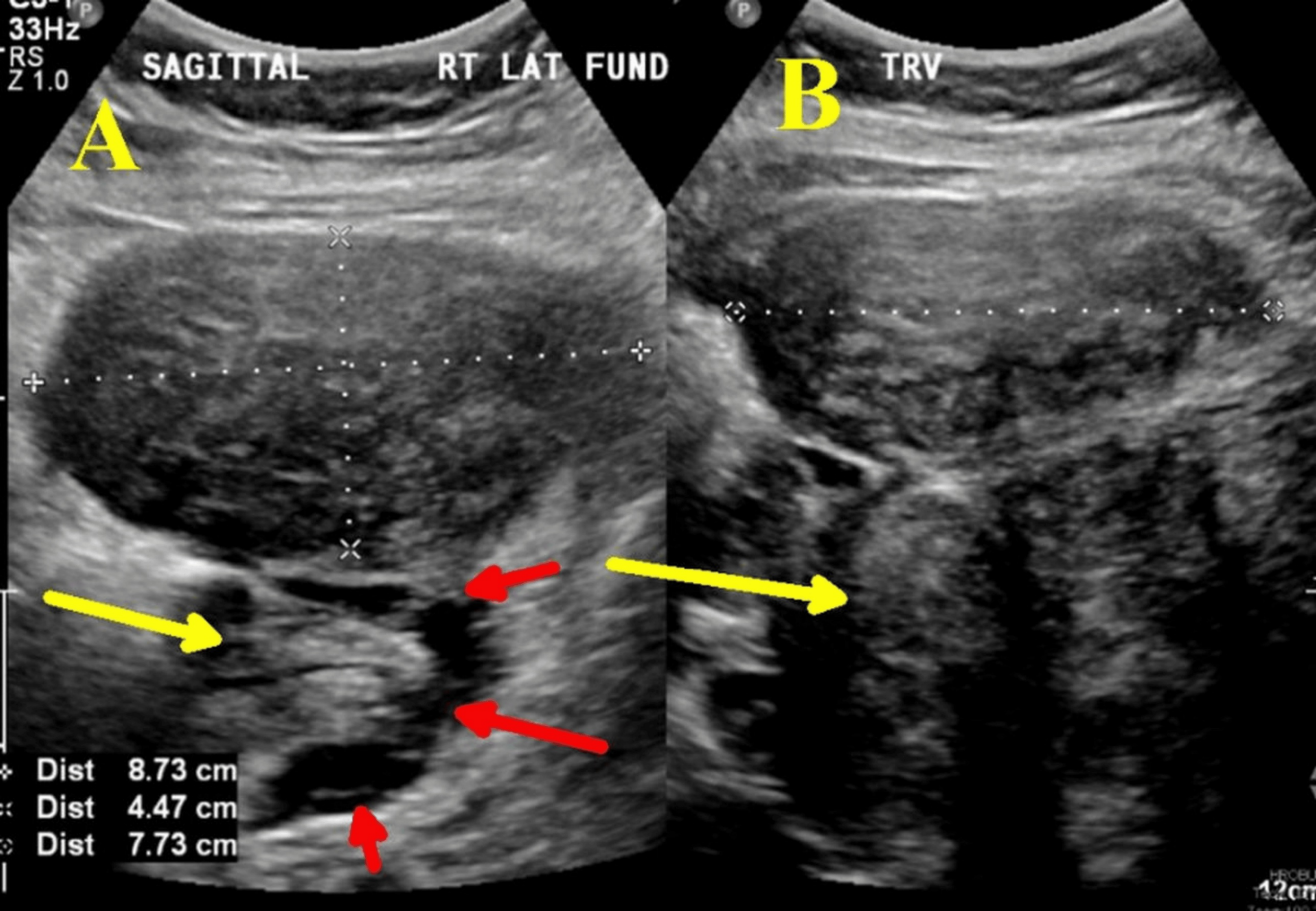
Obstetric ultrasound on admission at 23 weeks and 5 days Image A is a sagittal view, and image B is a transverse view. The yellow arrows on A and B show the fetus; the red arrows in image A show the amniotic fluid, and one of her myomas is between the hashmarks in images A and B, measuring 8.73 cm x 4.47 cm x 7.73 cm.

A 32-year-old woman (gravida 2 para 0) presented at 23 weeks and 5 days of gestation for preterm labor evaluation. She received her prenatal care outside of the United States and was diagnosed with cervical insufficiency. An Arabin pessary was placed nine days prior to admission to our hospital. It is unclear if membranes were visible on the speculum exam. She was initially seen at 20 weeks outside the United States and received indomethacin for ‘tocolysis’ because the cervix was noted to be funneling at 1.5 cm. On admission to our hospital, she was found to have PPROM confirmed by pooling of amniotic fluid and was noted to be 2 centimeters (cm) dilated. She did not complain of persistent uterine contractions. The cervical pessary was removed, and, after counseling, she was admitted for expectant management. Her medical history was negative, including no prior surgeries. For fetal lung maturity, she received betamethasone. She was also given latency antibiotics: intravenous ampicillin (2 grams every six hours for 48 hours), followed by oral amoxicillin (250 mg every eight hours). Additionally, she received one dose of azithromycin (1000 mg) and magnesium sulfate for fetal neuroprotection. An ultrasound performed at admission (Figure 1) showed a single fetus in cephalic presentation, with the deepest pocket of amniotic fluid measuring 5.2 cm and an estimated fetal weight of 606 grams.

Fetal monitoring indicated no contractions or signs of distress. The patient continued to experience fluid leakage but remained clinically stable, with normal results on the comprehensive metabolic profile (CMP) and no significant changes in laboratory values (Table 1) or vital signs (Table 2).

**Table 1:** Serial laboratory values from admission till day 78

Data on first admission	Normal Range	Admit, Day 1	Day 7	Cesarean, Day 15	Postoperative Day 2, Day 18 after first admission	Discharge, Postoperative Day 5, Day 20 after first admission	Re-admission #2, Postop Day 8, Day 23 after first admission	Postoperative Day 11, Day 26 after first admission	Discharge, Postoperative Day 14, Day 29 after first admission	Re-admission #3, Postop Day 30, Day 46 after first admission	Postoperative day 35, Day 50 after first admission	Discharge, Postoperative day 40, Day 55 after first admission	Re-admission #4, Postop Day 50, Day 65 after first admission	Hysterectomy, Postoperative Day 52, Day 67 after first admission	Post hysterectomy Day 8, Day 75 after first admission
Hemoglobin (g/dL)	Women -12.3 to 15.3 g/dL (grams per deciliter)	11.6	11.6	11.1	10.6	9.5	9.4	10.5	9	9.5	9.1	8.7	6.8	8.2	9.3
White blood cell (cells/µL)	4,500 - 11,000 cells/µL (cells per microliter)	9,700	11	16,900	8,900	11,600	15,200	11,400	7,700	12,600	12,300	10,900	13,200	9.8	7,000
Platelet ( x10^9^/L)	100,000 - 400,000/L (per liter)	1,32,000	1,35,000	1,09,000	94,000	1,28,000	2,15,000	3,37,000	3,29,000	2,15,000	2,22,000	2,97,000	3,66,000	3,88,000	3,69,000

**Table 2:** Serial vital signs from admission till day 78

Data on first admission	Normal Range	Admission, Day 1	Day 7	Cesarean, Day 15	Postoperative Day 2, Day 18 after first admission	Discharge, Postoperative Day 5, Day 20 after first admission	Readmission #2, Postoperative Day 8, Day 23 after first admission	Postoperative Day 11, Day 26 after first admission	Discharge, Postoperative Day 14, Day 29 after first admission	Readmission #3, Postoperative Day 30, Day 46 after first admission	Postoperative day 35, Day 50 after first admission	Discharge, Postoperative day 40, Day 55 after first admission	Readmission #4, Postoperative Day 50, Day 65 after first admission	Hysterectomy, Postoperative Day 52, Day 67 after first admission	Post hysterectomy Day 8, Day 75 after first admission	Discharge, Post hysterectomy, Day 11, Day 78 after first admission
Blood pressure	120/80 mm/Hg	124/71	100/62	101/60	121/56	123/76	155/74	118/68	120/75	106/67	109/59	115/67	108/73	119/73	127/80	104/55
Oxygen saturation	95 - 100%	99%	99%	100%	96%	98%	97%	98%	100%	99%	99%	100%	97%	100%	98%	99%
Heart rate	60-100 beats per minute (bpm)	104	96	92	124	89	93	80	66	67	104	69	88	85	84	61
Temperature	Farrenheit °	99.1	98.5	97.3	102.7	98.3	100.7	99.5	98.1	97.6	100.5	98.6	98.2	98.5	98	98.3

After 15 days of inpatient management, the patient reported contraction pain and pressure, and fetal heart tracing was significant for non-reassuring fetal surveillance consisting of decelerations and minimal variability. Because of ongoing fetal deceleration and non-reassuring status remote from delivery, an emergency CS was performed under regional anesthesia via Pfannenstiel incision using the classic technique. The classic vertical incision through the uterine muscle was chosen due to anatomical factors, including a bulky fibroid uterus with several pedunculated and intramural fibroids, notably a large anterior one near the lower uterine segment. Umbilical cord blood could not be obtained for metabolic status because of coagulation. The time from uterine incision to fetal delivery was five minutes, and multiple attempts to deliver the fetal head were necessary. The male infant weighed 800 gr, and the Apgar scores were 1 at one minute, 5 at five minutes, and 7 at 10 minutes. The neonate expired on the first day of life secondary to multiple complications, including sepsis and prematurity. Histopathological examination demonstrated funisitis extending into Wharton’s jelly, a placenta weighing 224 grams exhibiting inflammatory changes, and acute chorioamnionitis. Intraoperative intrauterine and membrane cultures were not performed.

The patient was diagnosed with septic chorioamnionitis and a urinary tract infection. Blood cultures identified* Escherichia coli*, while urine cultures yielded *Serratia marcescens* and *Pseudomonas aeruginosa*. She stabilized after postoperative day 2 and was discharged on postoperative day 5 (day 20 after the original admission). Intravenous (IV) metronidazole, 500 milligrams (mg) every eight hours, and ceftriaxone, 2 grams (g) each day, were initiated postoperatively, with planned discontinuation after four days and 10 days, respectively.

She returned three days later (postoperative cesarean day 8 and day 23 after the first admission) for admission number 2 with leukocytosis, a fever, abdominal pain, and shortness of breath. Guarding and tenderness were noted at the surgical site, but there was no incisional drainage. Speculum findings reflected a typical postoperative cesarean state, and the breast exam showed no infection.

**Figure 2:**
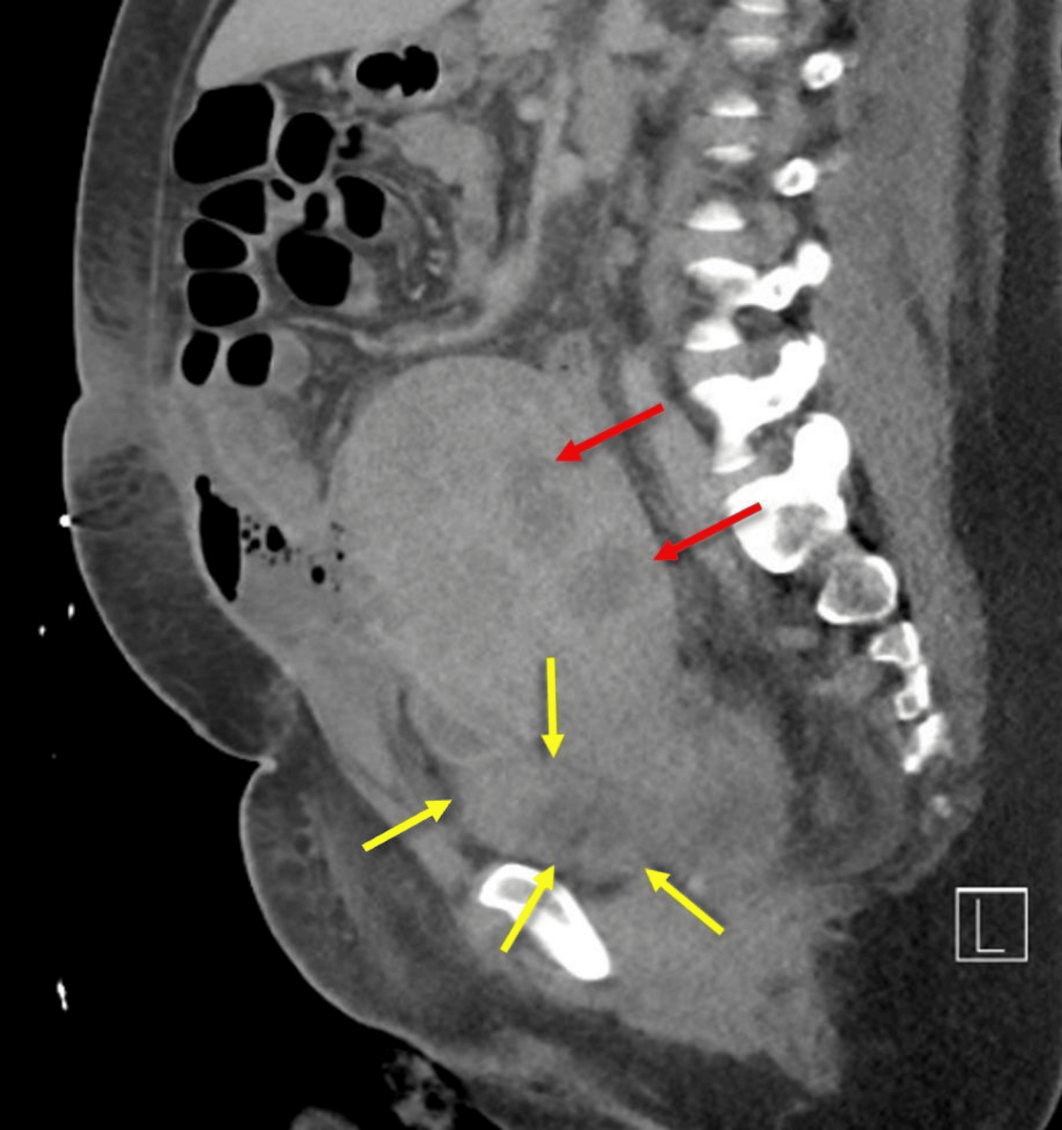
CT of the abdomen and pelvis (sagittal plane) The yellow arrows show fluid collection that is contiguous with the anterior uterine wall in the region of the surgical site. Intraperitoneal abscess, postoperative seroma, and resolving hematoma were all in the differential diagnosis. The red arrows show the nodularity in the uterine wall consistent with myomas. Intrauterine abscess and endometritis could not be excluded.

A workup excluded pulmonary embolism and cardiorespiratory causes. A CT suggested abnormal fluid collections consistent with an abscess (Figure 2); interventional radiology attempted drainage, but only minimal sterile fluid was obtained.

**Figure 3:**
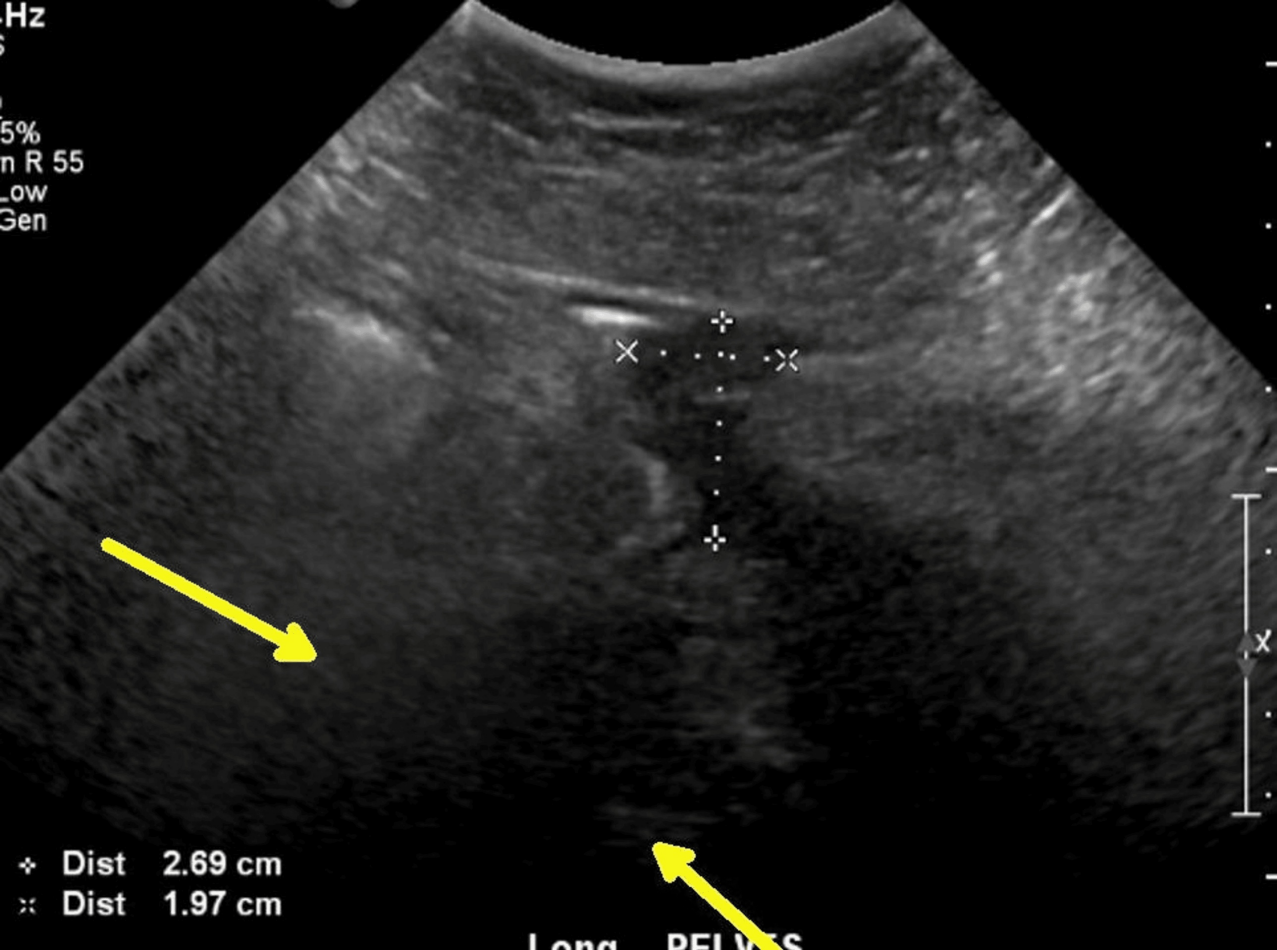
Transabdominal ultrasound (day 28 after initial admission and 13 days after cesarean delivery) showing the complex fluid collection anterior to the uterus between the cursors, measuring 2.69 cm x 1.97 cm. The region of the uterus is below the acoustic shadowing at the yellow arrows.

Piperacillin-tazobactam 4.5 g IV every six hours was started by the infectious diseases team; clinical improvement led to discharge after six days on postoperative cesarean day 14, day 29 after the first admission. A pelvic ultrasound conducted prior to discharge demonstrated a reduction in the size of the complex fluid collection, measuring approximately 3 cm and located anterior to the uterus at the site of the classic incision (Figure 3).

**Figure 4:**
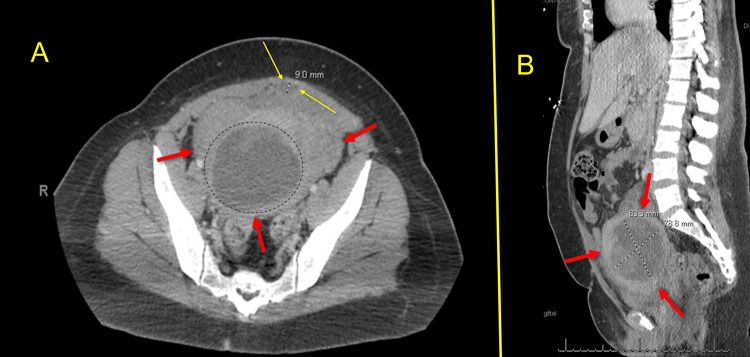
CT scan of the pelvis (transverse, image A) and abdominal/pelvic (sagittal, image B) (day 46 after initial admission and 30 days after cesarean delivery). The long yellow arrows in image A show a near resolution of the midline rim-enhancing fluid collection measuring less than 1 cm. Also, within the black hashed circle, there is a mild increase noted in the “cystic lesion within the uterus” (8.8 cm x 7.8 cm x 8.1 cm), suggestive of a necrosed fibroid versus retained products versus a uterine abscess. The uterine wall is indicated by the red arrows. Image B shows the mass within the uterus (hash marks), with the red arrows showing the uterine wall.

Sixteen days later (postoperative CS day 30 and day 46 following the initial admission), the patient was readmitted for a third time due to leukocytosis, persistent fevers, and abdominal wall wound tenderness. Her CMP was normal. She was treated with ceftriaxone 2g IV daily. A CT scan (Figure 4) demonstrated that the midline rim-enhancing fluid collection had nearly resolved, measuring approximately 0.9 cm.

**Figure 5:**
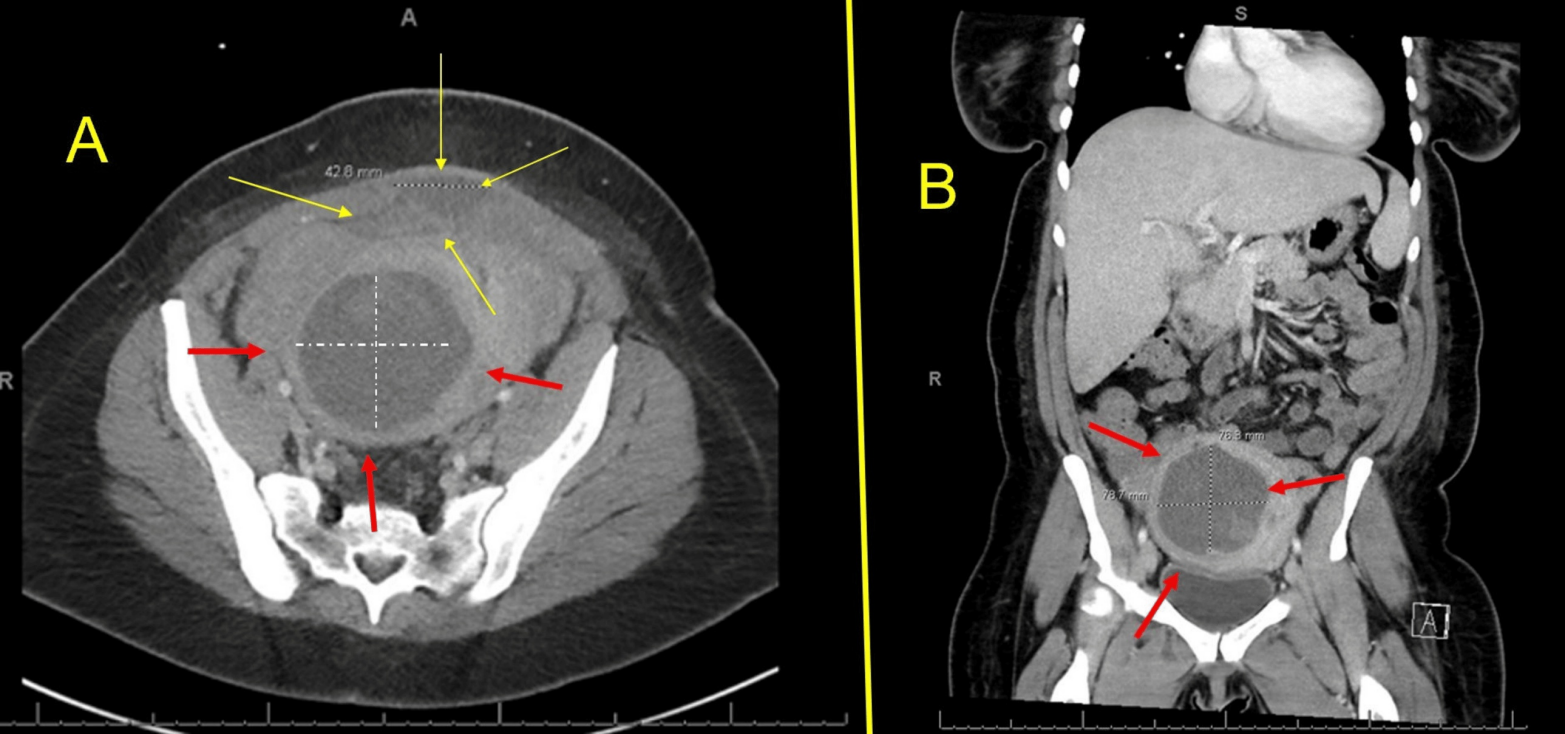
CT scans of the pelvis (transverse, image A) and abdominal pelvic (coronal, image B) (day 50 after initial admission and 35 days after cesarean delivery). The fluid collection in the uterus had decreased slightly to 7.6 cm x 7.9 cm (between hash marks in images A and B), and differential diagnosis included degenerated necrotic uterine fibroid versus myometrial abscess. Concern for the progression of an abscess in the anterior pelvic wall with an increase in all dimensions, measuring now 4.2 cm (long yellow arrows in image A). The red arrows in images A and B are the uterine wall.

Additionally, there was a slight increase in the size of a cystic lesion within the uterus (8.8 x 7.8 x 8.1 cm), raising consideration for a necrosed fibroid, retained products of conception, or a uterine abscess. Subsequent ultrasound imaging performed two days later measured the lower uterine segment mass at 7.9 x 7.2 x 7.6 cm, consistent with prior CT findings. Repeat aspiration of the uterine cystic lesion by interventional radiology yielded less than 5 mL of reddish fluid and cultured *Escherichia coli* (Table 1). Five days after her admission (postoperative CS day 35 and 51 days from her initial admission), a repeat CT scan was obtained to better delineate the fluid collections (Figure 5).

**Figure 6:**
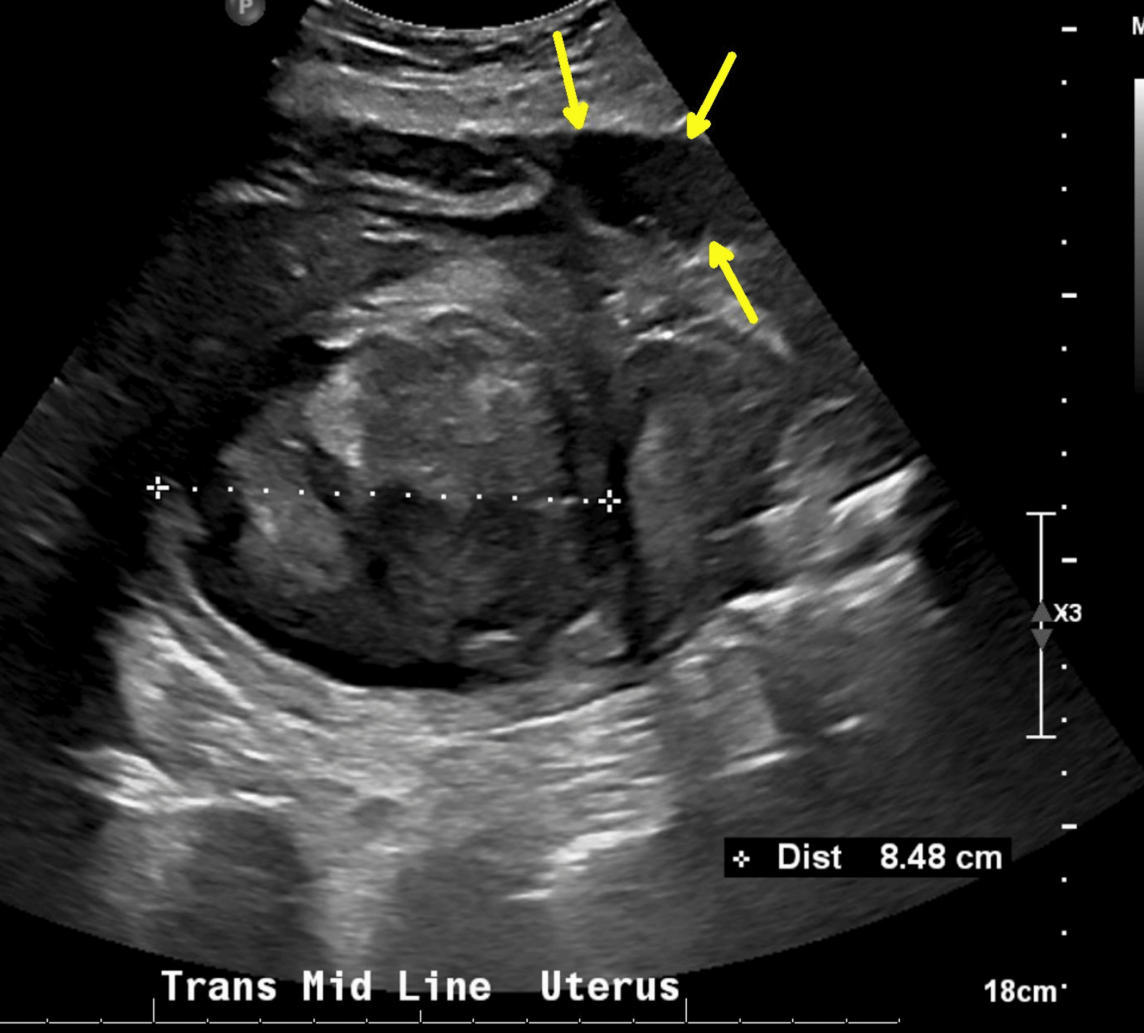
Transverse image of pelvic ultrasound (day 54 after initial admission and 39 days after cesarean delivery). The area between the yellow arrows represents fluid above the uterus in the region of the cesarean incision, measuring approximately 4 cm x 2.5 cm. The uterine wall with a myoma measures 8.48 cm and is between the cursors.

The intrauterine fluid collection showed a slight reduction in size. Differential considerations included a degenerated necrotic uterine fibroid versus an endometrial or myometrial abscess, with potential drainage impeded by the fibroid acting as an obstructive plug at the cervical os. Additionally, the CT scan demonstrated a possible increase in the size of a fluid collection between the abdominal wall and the uterine wall at the site of the cesarean incision, expanding from 0.9 cm to 4.2 cm transversely. Due to concerns regarding insufficient source control, surgical intervention was considered; however, the patient expressed a strong preference for preserving future fertility and consequently elected conservative management. She was discharged after 10 days (postoperative cesarean day 40 and day 55 following the original admission) with a regimen of intravenous ceftriaxone 2 g daily for 10 days and arrangements for outpatient wound care. Prior to discharge, a follow-up ultrasound (Figure 6) was performed to evaluate the status of intrauterine and anterior fluid collections.

Anterior fluid collection, measuring 4 cm, was again noted.

**Figure 7:**
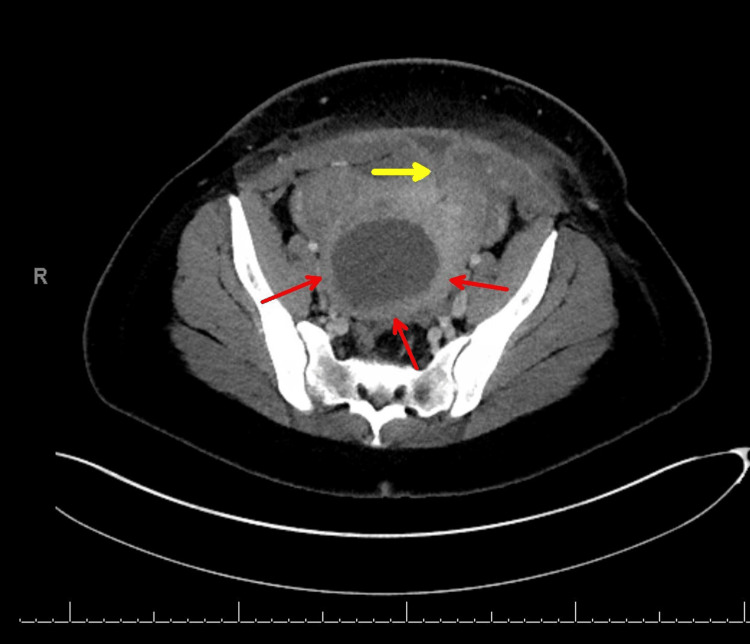
Transverse CT scan on the pelvis (day 65 after initial admission and 50 days after cesarean delivery). There is a persistent fluid collection in the left ventral abdominal wall concerning for an abscess (yellow arrow). Possible fistulous tract between the abdominal wall collection and myometrium, possibly in continuity with the uterine abscess, versus a degenerating fibroid. The uterine wall is indicated by the red arrows.

Eleven days later (postoperative cesarean day 50, day 65 from initial admission), the patient was admitted for the fourth time with worsening abdominal wall symptoms: incisional drainage, tenderness, erythema, and increased warmth. Speculum exam found moderate reddish-yellow discharge. She was afebrile but had leukocytosis (Table 1). CT showed minimal change in previous fluid collections, but new subcutaneous edema, fat stranding, and ventral abdominal wall thickening indicated cellulitis. A fistulous tract was seen between the abdominal wall collection and myometrium, possibly connecting to a uterine abscess or degenerating fibroid (Figure 7).

**Figure 8:**
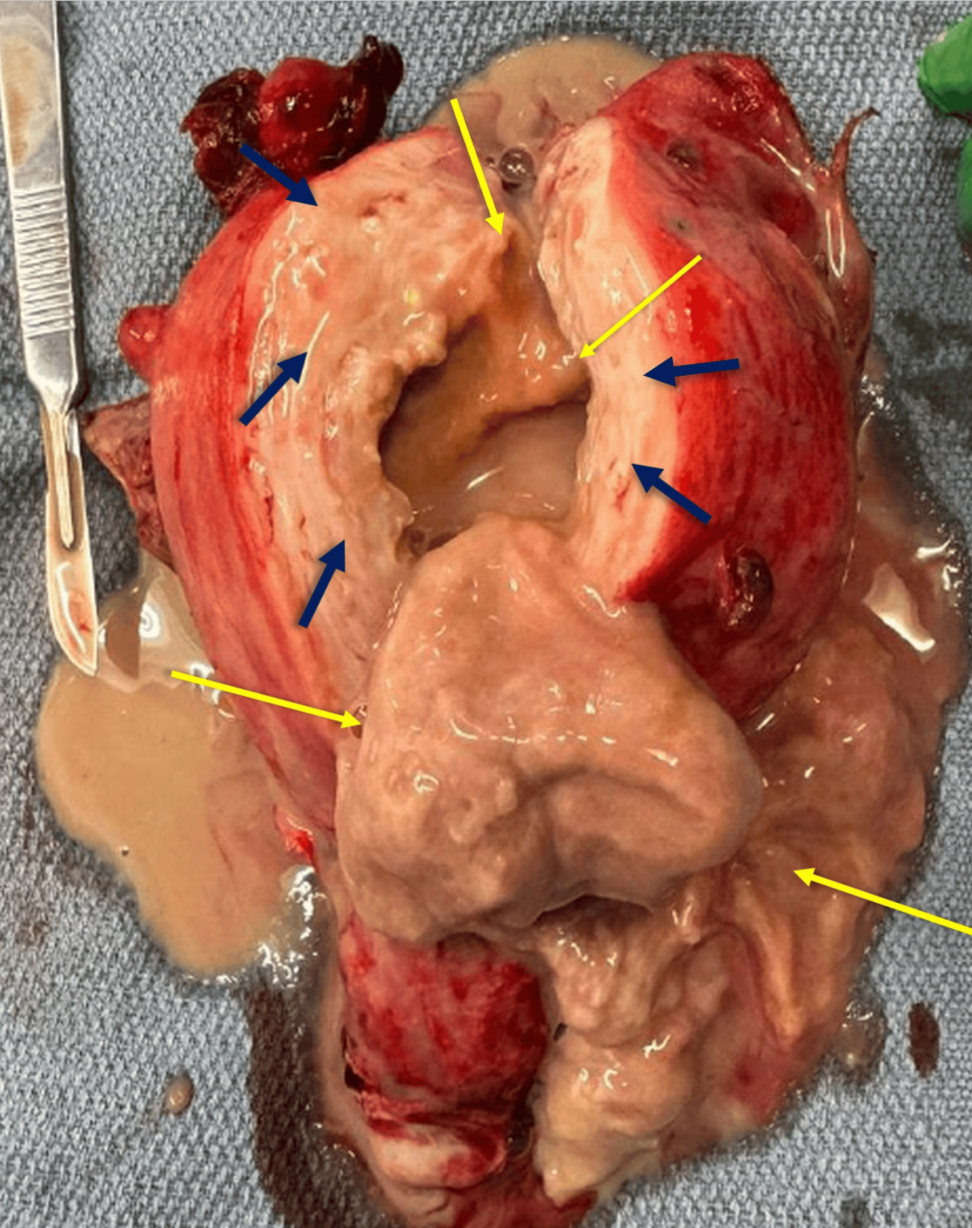
Pathology specimen of the uterus (day 67 after initial admission and 52 days after cesarean delivery). The dark blue arrows show tissue consistent with myomatous ischemia, involution, and degeneration. Purulent discharge is on the end of the knife's blade. The yellow arrows surround the abscess cavity and inflammatory mass.

The infectious diseases and general surgery teams were consulted again. Because the infection persisted and could not be adequately controlled, a consensus was reached to move forward with a hysterectomy. The patient was started on intravenous piperacillin-tazobactam (4.5 g every eight hours), vancomycin (500 mg IV, four times daily), and metronidazole (500 mg IV, every six hours). On day 67 following her initial admission and day 52 after the classic cesarean, she underwent a total abdominal hysterectomy with bilateral salpingectomy. Pathology findings included a uterus weighing 300 g containing a 6 cm cavitary abscess associated with suture material. Multiple degenerated fibroids up to 2.8 cm were found in the myometrium (Figure 8).

Her fallopian tubes showed both acute and chronic salpingitis, and no trophoblastic tissue was present. Postoperative recovery was smooth except for receiving a single-unit blood transfusion on the first day after surgery. She was later discharged home with a wound vacuum device applied.

At her six-week postoperative visit, the patient was noted to be recovering well and was cleared for all activities. Patient consent was obtained.

## Discussion

This case is significant for three main reasons: preterm delivery, infection, and coordinating serial radiological findings with clinical presentation and patient autonomy.

Her preterm delivery was due to cervical insufficiency, which likely caused her PPROM. Aside from multiple myomas, she had no other obstetric risks for cervical insufficiency [5]. Cerclage and vaginal progesterone are effective management options [6]. The Arabin pessary, introduced in the 1970s as a cerclage alternative, has shown limited benefit in preventing preterm birth among high-risk patients, according to larger studies, despite some small studies suggesting efficacy [7-9].

The relationship between myomas and the latency interval from PPROM to delivery remains unclear [10]. In this case, the patient's latency period was 15 days, which aligns with reported PPROM latency intervals at 23 weeks, typically ranging from one to three weeks [11]. The onset of her labor after 15 days was most likely due to infection, as evidenced by the neonatal condition and histopathological findings of the placenta and membranes. Inflammation and amniotic infection are commonly identified as primary causes of labor following PPROM [12].

The second important consideration in this case pertains to infection-related findings. Conducting a cesarean section in the setting of chorioamnionitis substantially increases the likelihood of complications such as endometritis, wound infections, and pelvic abscesses [13]. Furthermore, systematic review and meta-analysis results demonstrate that classic cesarean deliveries performed between 26 and 31 weeks of gestation are associated with a higher incidence of endometritis [14]. In alignment with established literature, our case revealed that antibiotic therapy alone was inadequate for resolving a postoperative abscess, necessitating surgical drainage [15]. The patient presented with two distinct purulent collections: one intrauterine, likely undrained due to obstruction by a myoma resulting in encapsulation, and another adjacent to the classic incision and overlying abdominal wall, which subsequently drained via a fistulous tract prior to her final admission. Both abscesses were closely observed through imaging studies, and interventional radiology-guided drainage was attempted. The ultimate diagnosis included abscess formation of the surgical wound, degenerating uterine myomas, and involvement of the uterine cavity.

The third important element to this case is coordinating the clinical findings with imaging and patient directives over a two-month postoperative period. She wished to preserve her fertility despite evidence of persistent infection affecting her myomas, endometrial cavity, surgical wound, or all three. Antibiotic therapy kept her stable, allowing for delayed surgical intervention. This case highlights the challenges and limitations of imaging in managing complex postpartum infections, particularly with fibroid uteri and possible postoperative intra-abdominal or pelvic infections [16,17]. It is well-established that imaging a postpartum uterus routinely is not reliable secondary to distortions as a result of normal postpartum changes [18]. It was more challenging in this case because of the additional factors of a post-classical cesarean with myomas in a preterm delivery that was characterized by a 52-day postoperative course with slowly progressive myoma degeneration and surgical site infection. Although imaging was instrumental in multiple phases of care for this patient, it highlighted diagnostic uncertainty that can arise when interpreting findings secondary to distorted postoperative anatomy and progressive changes over time [19]. The fluid collected between the uterus and the anterior abdominal wall was difficult to define over time and between imaging modalities. It varied between .9 and 4.0 cm. The uterine cystic structures that ultimately were found to be abscesses were confusing between red degeneration and abscess, especially when interventional radiology could not drain the sac. Serial imaging during her subsequent hospitalizations demonstrated fluid collections adjacent to or within degenerating myomas. Degenerating myomas, particularly those undergoing red degeneration, can appear as mixed-density masses on CT, mimicking abscesses [20]. This overlap in radiologic appearance complicates differentiation between post-surgical hematoma, infected fibroid degeneration, and true abscess formation. Furthermore, the distorted anatomy due to multiple pedunculated and intramural myomas made it more difficult to accurately localize fluid collections and assess for progression or resolution over time. Notably, during this hospitalization, the image-guided aspiration of the presumed degenerating fibroid yielded *Escherichia coli *growth, indicating that possibly the fibroid itself had become the nidus of persistent sepsis.

Serial imaging throughout hospitalization showed evolving but nonspecific findings, and the lack of clear improvement despite antibiotic therapy and image-guided drainage increased suspicion for inadequate source control. The final imaging prior to hysterectomy demonstrated persistent pelvic fluid collections and inflammatory changes despite medical management. These findings, combined with clinical deterioration, helped guide the patient to permit surgical intervention. Ultimately, definitive source control was achieved only through total abdominal hysterectomy, confirming the suspected infected degenerating myomas, intrauterine abscess cavity, and wound space as the likely ongoing source of infection.

## Conclusions

A classic cesarean delivery in a preterm pregnancy complicated by multiple myomas and chorioamnionitis is extremely high risk for maternal and infant complications. Radiologic imaging played a central role in the diagnosis and management of this complex postpartum course. However, its limitations became evident with the distorted pelvic anatomy and overlapping postoperative changes. While CT and ultrasound provided critical guidance for drainage attempts and monitoring, they were ultimately insufficient to help distinguish between degenerating fibroids and infected abscesses. This highlights the importance of integrating radiologic findings with clinical presentation and laboratory findings to guide management. Repeated imaging without improvement should raise concern for persistent infection despite nonspecific radiologic appearances, as in this case. Ultimately, while imaging can help inform patient management, it cannot dictate the clinical decision-making for high-acuity patients, such as in the care of a postpartum patient with complex uterine anatomy, fibroid-associated infection, and persistent sepsis.
